# Prenatal Genetic Testing for Dopa-Responsive Dystonia – Clinical Judgment in the Context of Next Generation Sequencing

**DOI:** 10.25122/jml-2018-0076

**Published:** 2018

**Authors:** Florina Nedelea, Alina Veduta, Simona Duta, Ana-Maria Vayna, Anca Panaitescu, Gheorghe Peltecu, Hans-Christoph Duba

**Affiliations:** 1. Filantropia Clinical Hospital, Bucharest, Romania; 2. Carol Davila University of Medicine and Pharmacy, Bucharest, Romania; 3. Kepler Universitätsklinikum Linz, Austria

**Keywords:** prenatal diagnosis, Next Generation Sequencing, dystonia

## Abstract

We present a family in which the first child was diagnosed with dopa-responsive dystonia based on biochemical findings only. Dopa-responsive dystonia is a severe heterogeneous genetic disease. The possibly involved genes are GCH1 and TH.

In their second pregnancy, the parents came for genetic counseling and prenatal diagnosis late, at 12 weeks of gestation. Genetic testing in the affected child was performed, but the results were difficult to interpret. The identified mutations were classified as VOUS – variants of unknown clinical significance. Although possibly causative, a homozygous variant in the TH gene was not reported before in children with dopa-responsive dystonia. Due to limited time, establishing the fetal prognosis was challenging.

Our report emphasizes the importance of a multidisciplinary approach in the context of new diagnostic techniques, such as Next Generation Sequencing. We illustrate the fact that behind any laboratory result remains sophisticated clinical judgment. We also describe a previously not reported variant of the TH gene in a child with severe, early-onset dystonia.

## Introduction

Prenatal diagnosis in families with children affected by genetic syndromes is essential but can be time-consuming because of the heterogeneity of some genetic disorders and because of difficulties in interpreting genetic results. Preconception genetic counseling is the optimal approach in subsequent pregnancies; otherwise, the time needed for prenatal diagnosis might become an issue.

Dopa-responsive dystonia is a heterogeneous genetic disease. The genes GCH1 and TH are known to be involved [1–2]. Mutations in the GCH1 gene are responsible for the disease beginning in childhood, around six years of age [1]. There is remarkable responsiveness to levodopa (L-dopa), in over 80% of cases. Mutations in the TH gene are responsible for the disease presenting early, in the first year of life, with a progressive hypokinetic-rigid syndrome and generalized dystonia. Less frequently, a more severe phenotype of complex encephalopathy can present before the age of 6 months [1].

## Clinical Report

A 32-year old patient sought advice on prenatal genetic diagnosis at 12 weeks of gestation, in her second pregnancy. She was referred for genetic counseling and invasive prenatal testing because she had a child with dopa-responsive dystonia. The diagnosis was made during the child’s first year of life, based on biochemical findings. The affected boy was the first child of healthy, not related parents; he was three years and eight months old at the time we saw the couple, in their second pregnancy. Familial history revealed no other affected cases. The conventional first-trimester screening for aneuploidy in the on-going pregnancy indicated a low risk.

**Figure 1:**
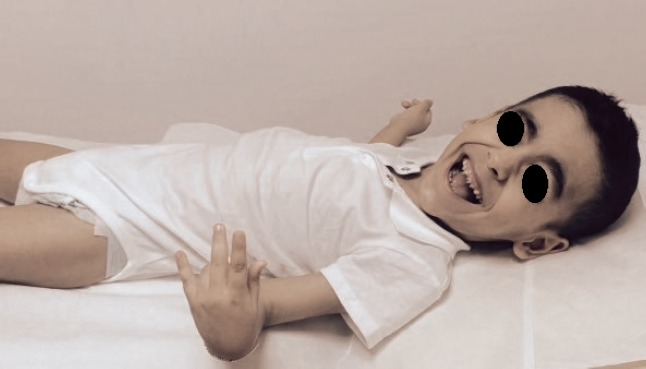
Child affected by severe dopa-responsive dystonia. Note the dysmorphic features, ptosis and limb spasticity.

The child presented with dysmorphic features, ptosis, truncal hypotonia, limb spasticity, oculogyric crises and severe developmental delay (Figure1). The therapy with levodopa was interrupted by the family because of adverse effects and lack of response. No genetic testing had been done, and the causative genetic mutation was not known; therefore, chorionic villus sampling could not be performed in the on-going pregnancy.

Our approach was to begin genetic testing in the affected child. Although the clinical picture correlates better with TH deficient dopa-responsive dystonia, due to limited time and broad variation of the clinical phenotype, Next Generation Sequencing (NGS) was chosen as the method of analysis for both possibly involved genes. NGS is a powerful diagnostic tool; still, we were aware that difficulties could arise in correlating NGS results and a specific clinical phenotype [3–4]. Taking into account all advantages and being aware of possible results, we decided for genetic diagnosis using NGS which, in our case, had a turnaround time of five weeks for the affected child.

NGS identified a heterozygous variant c.671A>G (p.Lys224Arg) in the GCH1 gene; this has been previously described in patients with dopa-responsive dystonia [5–6]. The variant is reported in dbSNP (rs41298442; MAF 0.04), ESP (0.02%) and gnomAD (0.036%, 100 heterozygotes reported) [8–10]; it is located in a highly conserved residue, and bioinformatics analysis is not conclusive about its pathogenicity. It was classified as a variant of unknown clinical significance, VOUS, in the original report.

An apparently homozygous variant c.1389C>G (p.Phe463Leu) in the TH gene was also detected. This variant is not reported in the literature or the dbSNP, ExAC and ESP databases. It is located in a highly conserved residue, and bioinformatics analysis considers it to be possibly damaging. Based on the available information, this should also be classified as a variant of unknown clinical significance, VOUS. Mutations in the TH gene are responsible for autosomal recessive Segawa syndrome (OMIM 605407) [11] (as stated in the original report). Additional deletion/duplication analysis of GCH1 and TH genes using MLPA was performed, and no deletions/duplications were detected in the analyzed regions.

The parents requested prenatal diagnosis, and an amniocentesis was performed at 18 weeks of gestation.

We were therefore faced with a difficult situation: genetic analysis without a compelling genetic cause and limited time to make the decisions regarding prenatal testing. Our judgment was not to perform prenatal genetic testing for the mutation in the GCH1 gene only. The GCH1 deficient phenotype typically becomes obvious at about six years of age, and even in the context of clinical variability, the identified mutation was unlikely to be causative in this case. Moreover, as for autosomal dominant conditions in general, the mutation in affected individuals are de novo mutations most often, and the risk of recurrence is very low.

Mutations in TH gene can be responsible for the recessive form of the disease, which may present with the severe form of TH deficient progressive infantile encephalopathy. This better correlated with our case and the recurrence risk in the on-going pregnancy would have been high. Unfortunately, the detected homozygous mutation in the TH gene was classified as a variant of unknown clinical significance; according to the prenatal testing guidelines available [4], testing of the fetus would generally not be advised in such cases. In this specific context, the parents have been informed about the meaning and the possible implications of the result. Because of the significant clinical correlations and severity of consequences, the parents decided to have an amniocentesis and to test the fetus. The genetic result indicated a heterozygous TH mutation in the fetus and the pregnancy continued. A clinically normal child was delivered at term, and early postnatal evolution was good.

## Discussion

In the case of this family with a boy affected by dopa-responsive dystonia, the limited available time and the genetic heterogeneity of the disease made prenatal diagnosis challenging. NGS identified mutations of both GCH1 and TH genes in the affected child, while the mother was pregnant again. Both mutations were classified as variants of unknown significance, VOUS. The homozygous variant found in the TH gene, c.1389C>G (p.Phe463Leu) was never before reported in children with dopa-responsive dystonia. Under the circumstances, deciding which genetic tests to perform for the fetus was an intricate matter of medical judgment and parental choice.

Although genetic testing options have evolved massively, the process of genetic diagnosis should be strongly correlated with clinical phenotype. Multidisciplinary approach and collaboration with clinical genetics are strongly recommended in difficult familial cases.

## Conclusion

The recurrence risk in families with known genetic diseases should be assessed preconceptionally, in order to allow time and resources for prenatal diagnosis and optimal pregnancy care.

## Conflict of Interest

The authors confirm that there are no conflicts of interest.

## References

[1] Furukawa Y, Guttman M, Nakamura S, Kish SJ, Frucht SJ (2013). Dopa-responsive dystonia. *Movement Disorder Emergencies: Diagnosis and Treatment*.

[2] Furukawa Y (2004). Update on dopa-responsive dystonia: locus heterogeneity and biochemical features. *Adv Neurol*.

[3] Thiffault I, Lantos J (2016). The challenge of analyzing the results of Next Generation Sequencing in children. *Pediatrics*.

[4] Richards S, Aziz N, Bale S, Bick D, Das S, Gastier-Foster J, Grody WW, Hegde M,, Lyon E, Spector E, Voelkerding K, Rehm HL (2015). Standards and guidelines for the interpretation of sequence variants: a joint consensus recommendation of the American College of Medical Genetics and Genomics and the Association for Molecular Pathology. *Genet Med*.

[5] Leuzzi V, Carducci C, Carducci C, Cardona F, Artiola C, Antonozzi I (2002). Autosomal dominant GTP-CH deficiency presenting as a dopa-responsive myoclonus-dystonia syndrome. *Neurology*.

[6] Bandmann O, Nygaard TG, Surtees R, Marsden CD, Wood NW, Harding AE (1996). Dopa-responsive dystonia in British patients: new mutations of the GTP-cyclohydrolase I gene and evidence for genetic heterogeneity. *Hum Mol Genet*.

[7] Bandmann O, Wood NW (2002). Dopa-responsive dystonia – the story so far. *Neuropediatrics*.

[8] Short Genetic Variations Database (dbSNP) https://www.ncbi.nlm.nih.gov/SNP/snp_ref.cgi?rs=rs41298442.

[9] Exome Sequencing Project (ESP) http://evs.gs.washington.edu/EVS/ServletManager?variantSNPSummary=Display+SNP+Summary.

[10] Genome Aggregation Database (gnomAD) http://gnomad.broadinstitute.org/variant/14-55310817-T-C.

[11] Online Mendelian Inheritance in Man https://www.omim.org/entry/605407.

